# Some Interesting Canine Cases

**Published:** 1896-04

**Authors:** Cecil French

**Affiliations:** D.V.S. (McGill and Munich), the Washington (D. C.) Canine Infirmary


### REPORTS OF CASES.

### SOME INTERESTING CANINE CASES.

BY CECIL FRENCH, D.V.S. (McGILL AND MUNICH),
THE WASHINGTON (D. C.) CANINE INFIRMARY.

*Suppurative Metritis in a Bitch—Diplococcus Lanceolatus in Pus.* A skye-terrier bitch, of light weight, six years of age, was brought to the Infirmary with little history other than that she had always enjoyed excellent health, had been safely delivered of three litters (the last some three years ago), but for a few weeks recently had been drooping. During her periods of œstrum a flow of blood was always very apparent. I mention this point because in another more recent case (which I hope to report as soon as its microbic nature has been determined) hemorrhage was markedly concurrent with the œstrual period.

Anorexia, frequent vomiting, constipation, subnormal temperature, and quick, weak pulse were manifested. The abdomen was pendulous and on percussion exhibited evidence of the presence of a large quantity of fluid in the uterine region.

It was decided to perform the operation of hysterectomy as the only chance for cutting short the septicæmia and prolonging life. The animal, however, did not survive the operation many hours.

Both cornua of the uterus were greatly distended, having a diameter of 4.5 cm. at the widest part, and contained a thick sanguineous pus, intercommunicating, which gave off a foul odor. The lumen of the body of the organ was completely obliterated by cicatricial tissue near the os, so that the contained matter had no means of escape. The Fallopian ducts were normal, so that the condition could hardly be defined as pyosalpinx. The mucosa was extremely ulcerated and thickened, showing that the condition had been a chronic one.

Macroscopic examination of the other organs showed no signs of pyæmia.

Professor Adami, of the McGill Pathological Laboratory, very kindly supplied the microscopic features of the case. Cover-slips of the pus stained in the usual manner showed the presence of a single form of micro-organism, a diplococcus definitely encapsulated, individuals varying from almost true coccus form to lanceolate shape, the appearance strikingly resembling that of *Diplococcus lanceolatus*, characteristic of pneumonia in man, and found also in a very large number of cases of suppurative diseases of various organs in man. Gameléia and Kinyoun, here in Washington, have both produced fibrous pneumonias in dogs by experimental inoculations (intrapulmonary) of this micro-organism. Sections of the uterine wall also showed the presence of this diplococcus.

*The Breath of the Dog Injurious to Health.* Dr. B., a practising physician of Washington, attributes the cause of a chronic condition of coryza, from which he formerly suffered, to inhalation of the breath of a pet dog which, to the sense of smell, was perfectly sweet.

The dog was accustomed to sleep on his bed in proximity to his head, and on awakening in the morning he would often experience a congested condition of the Schneiderian membrane, which was followed by the usual symptoms of ordinary cold in the head. One day the dog was sent away with the doctor's wife, whereupon the symptoms ceased to be manifested. Later, the gentleman rejoined his spouse, and on fraternizing with the dog under similar conditions, a return of the affection occurred. By further experiments he was convinced that the pulmonary exhalations of his dog were the sole cause of the trouble.

The case is interesting, inasmuch as it is the opinion of physiological investigators that the function of the lungs is not confined merely to the excretion of carbonic dioxide, but to the elimination of certain deleterious organic matters in highly attenuated form—that they are, in fact, important excretory glands, and that the vitiation of the atmosphere in crowded buildings is due not so much to the carbonic acid given off as to the dissemination of these poisonous ptomaïnes.

Foster states that an atmosphere of 1 per cent. of carbonic acid has little effect on the animal economy, whereas an atmosphere in which the carbonic acid has been raised to 1 per cent. by breathing is highly injurious.

*Longevity of Skye-terriers.* Mr. F. W., an engineer of this city, possesses a skye-terrier bitch that has attained the age of twenty-two years. She is still in excellent condition, and gives no promise of an early demise. A daughter of the same animal recently died aged eighteen years.

*Treatment of Strychnine-poisoning by Hypodermic Injection of Chloral Hydrate.* Having found the oral administration of chloral hydrate an impossibility and the rectal injection of the same drug usually too slow in action to be of any benefit in the treatment of strychnine-poisoning, I have in two recent cases administered the drug by hypodermic injection, which was followed by prompt and satisfactory results. In each case a subcutaneous abscess subsequently formed at the point of injection, but both responded to proper treatment and quickly healed.

Authorities on therapeutics teach that this drug is too irritating to be administered hypodermically. There can be no question, however, that in emergency cases it is far wiser to give it in this manner and treat the subsequent abscess than it is to risk losing a patient by consideration of instituting a superficial ailment easy of control.

